# Left Behind in Lockdown: How COVID-19 Deepened the Crisis in K-12 Physical Education

**DOI:** 10.3390/children12050603

**Published:** 2025-05-05

**Authors:** Da’Shay Templeton, Ruslan Korchagin, Bree Valla

**Affiliations:** Educational Leadership Department, Graduate School of Education, California Lutheran University, 60 W Olsen, Thousand Oaks, CA 91360, USA; rkorchag@callutheran.edu (R.K.); bvalla@callutheran.edu (B.V.)

**Keywords:** physical education, COVID-19 pandemic, physical fitness, social ecological model, educational equity

## Abstract

Background/Objectives: The COVID-19 pandemic has notably disrupted K–12 education globally, significantly impacting physical education and student health outcomes. This qualitative study investigates how the pandemic affected student physical fitness, motivation, and equitable access to fitness opportunities, particularly from the perspective of physical education teachers. Guided by the Social Ecological Model, the research addresses how intrapersonal, interpersonal, organizational, community, and policy factors collectively influenced student physical fitness outcomes post-pandemic. Methods: A phenomenological methodology was employed, utilizing semi-structured interviews conducted via Zoom with eleven K–12 physical education teachers in Ventura County, Southern California. Participants were selected using criterion sampling, targeting educators experienced in teaching before, during, and after the pandemic. Thematic analysis with initial in vivo coding was used to authentically capture participant perspectives, supported by strategies like peer debriefing and member checking to enhance analytical rigor. Results: Findings highlighted significant declines in student physical fitness post-pandemic, including reduced endurance, flexibility, and strength, accompanied by increased sedentary behaviors. Teachers reported pronounced decreases in student motivation and engagement, with heightened resistance to structured physical activities. Socioeconomic disparities deepened, disproportionately impacting economically disadvantaged students’ access to fitness opportunities and nutrition. Additionally, physical education experienced systemic undervaluation, resulting in inadequate resources, inconsistent policy enforcement, and difficulties in accurately assessing students’ fitness levels. Conclusions: This study underscores the urgent necessity for systemic reforms to rejuvenate physical education programs and promote equitable health outcomes. Recommendations include increased funding, improved facilities, reduced class sizes, consistent policy enforcement, and enhanced administrative support.

## 1. Introduction

The COVID-19 pandemic has profoundly reshaped multiple dimensions of daily life, significantly disrupting education systems globally. Among the areas most impacted is physical education, which plays a critical role in supporting physical fitness and overall student health [1]. The unprecedented shift from structured school environments to home-based remote learning has led to substantial declines in students’ physical fitness levels, motivation, and participation in regular physical activities [2,3]. Furthermore, socioeconomic disparities have intensified during this period, restricting access to quality physical education programs and organized sports for economically disadvantaged youth [4,5].

This article aims to explore how the COVID-19 pandemic has specifically affected the physical fitness levels of K–12 students, as perceived by physical education teachers. This research addresses the gap that was found in the literature: the perspective of the physical education teacher on the impacts of COVID-19. The qualitative approach to analyzing these practitioners’ experiences sheds a new perspective on how students fared before, during, and after the pandemic. Utilizing the Social Ecological Model as a theoretical framework, this investigation also examines how intrapersonal, interpersonal, organizational, community, and policy factors interact to influence student physical fitness outcomes in a post-pandemic context [6]. Additionally, the study seeks to understand the ways socioeconomic disparities have shaped students’ access to physical education and fitness opportunities following the pandemic. Through qualitative insights from educators, the article provides a comprehensive analysis of the challenges and barriers encountered, highlighting the necessity for targeted systemic reforms to rejuvenate physical education programs and promote equitable health outcomes for all students.

We sought to answer the following research questions: (1) How has the COVID-19 pandemic affected the physical fitness levels of K–12 students, as perceived by physical education teachers? (2) How do intrapersonal, interpersonal, organizational, community, and policy factors interact to shape student physical fitness outcomes in the post-pandemic context? (3) In what ways have socioeconomic disparities influenced students’ access to physical education and fitness opportunities post-COVID-19? The study revealed several critical insights into how the COVID-19 pandemic has affected physical education, as perceived by K–12 teachers. Educators observed a substantial decline in students’ overall physical fitness, with noticeable decreases in endurance, flexibility, and strength, accompanied by increased sedentary behaviors and weight gain. Alongside these physical changes, there was a pronounced reduction in student motivation and engagement, with many students displaying reluctance or outright refusal to participate in structured physical activities, preferring passive or social interactions instead.

Teachers further reported increased difficulties related to student compliance with participation requirements, such as dressing out, largely due to inconsistent enforcement of school policies. Socioeconomic disparities emerged as another significant concern, with economically disadvantaged students experiencing greater challenges accessing structured fitness opportunities, nutritious food, and extracurricular sports, which deepened pre-existing inequalities. The pandemic also intensified the devaluation of physical education within educational settings. Physical education programs often received insufficient administrative and governmental support compared to core academic subjects, resulting in inadequate funding, resources, and prioritization. Additionally, educators faced significant barriers in accurately assessing student fitness levels due to inconsistent student participation and the need to lower assessment standards to accommodate the diminished physical capabilities of students.

Overall, educators emphasized the critical need for additional resources and institutional backing to effectively address these post-pandemic challenges. They advocated for increased funding, improved equipment, smaller class sizes, additional staffing support, and stronger administrative and policy-level commitment to physical education, underscoring the necessity for comprehensive reforms aimed at restoring and sustaining equitable fitness education opportunities for all students. Implications for policy, research, and practice are discussed. The article is divided as follows: theoretical framework, literature review, methodology, findings, discussion, and conclusion.

### Theoretical Framework: Social Ecological Model of Health Behavior

**Figure 1:**
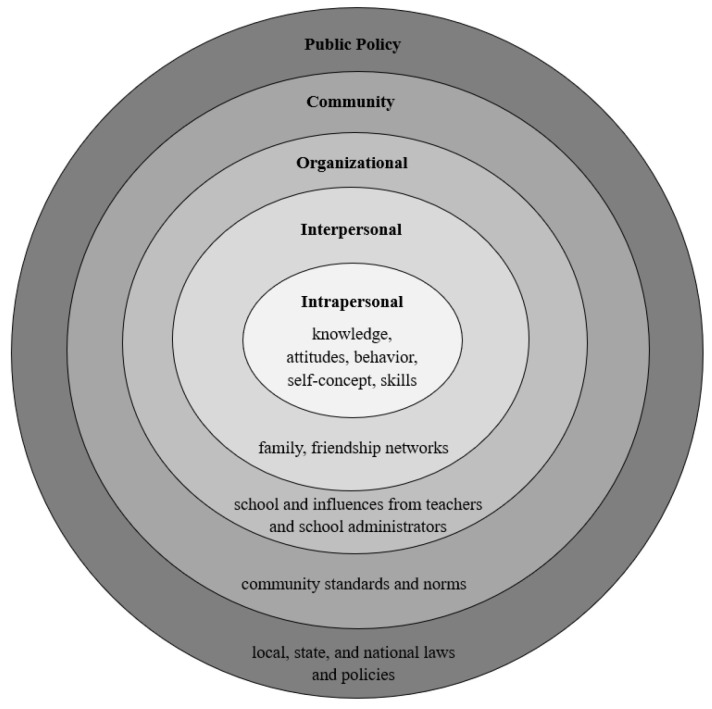
Social Ecological Model of Health Behavior. Note: This model, adapted from McLeroy et al. [6], illustrates the five levels of influence on individual behavior.

The Social Ecological Model is a theoretical framework that explains human behavior and health outcomes through the interaction of multiple environmental and social factors. The Social Ecological Model is based on the ecological systems theory developed by Bronfenbrenner [7]. Later, McLeroy et al. [6] formulated the Social Ecological Model and adapted it for public health. According to the Social Ecological Model, physical activity behavior is influenced by five levels or groups of factors: (1) intrapersonal factors, (2) interpersonal factors, (3) organizational factors, (4) community factors, and (5) public policy factors. Figure 1 shows groups of factors included in each level.

The Social Ecological Model can help explain how COVID-19 disrupted physical education in several ways, including home environments, school policies, and socioeconomic disparities. Using the Social Ecological Model helps examine barriers to student participation in physical education post-pandemic by considering student motivation (individual level), teacher influence (interpersonal level), school resources (institutional level), community access to sports (community level), and state/national education policy (policy level).

## 2. Literature Review

### 2.1. Decline in Physical Fitness Post-Pandemic

Physical fitness is crucial for maintaining overall health and well-being, including reducing the risk of cardiovascular disease, metabolic disorders, and mortality [8]. The benefits of physical activity include the prevention of chronic diseases, improvement in the health and function of the brain, promotion of mental health, and reduction in falls and injuries associated with them [9]. The COVID-19 pandemic has resulted in significant challenges for maintaining optimal physical fitness levels, resulting in an increase in sedentary behavior and a decrease in exercise participation among many individuals [1]. In the aftermath of the COVID-19 pandemic, acute weight gain has been reported, along with a reduction in physical activity and exercise and an increase in sedentary behavior [1,2]. Following the pandemic-induced lockdown, several dimensions of physical fitness were significantly undermined, including aerobic and anaerobic capacities, explosive power, and weight [3]. Several chronic conditions, including cardiovascular disease and type 2 diabetes, as well as premature mortality, are linked to these changes [10]. Additionally, the COVID-19 pandemic heightened anxiety, depression, and other psychological outcomes, such as worry and grief in children and older adults [11].

### 2.2. Lack of Motivation and Engagement and Challenges with Participation and Compliance

Even prior to the COVID-19 pandemic, many young people did not engage in the recommended amount of physical activity [12]. Children and adolescents should exercise at least 60 min each day to achieve necessary health benefits [13]. Also, three days per week should be spent exercising vigorously and strengthening muscles and bones. Even though physical education has great potential for promoting physical activity engagement, it is frequently considered to have a limited impact because of the limited curriculum time provided to the subject and ineffective practices that have traditionally had a narrow range of focus, delivered within a highly performative environment [14,15]. Social restrictions prevent students from achieving the necessary competencies in physical education. As an example, teachers in the United States are struggling due to a decline in student enthusiasm for physical education [16]. The lower motivational levels could, however, be related to the methods that teachers applied during the pandemic, which tended to be repetitive and routinized [17].

During the pandemic, students’ motivation to exercise was affected by different parts of their environments [18]. The home became a key place for activities, making parents and families more important in encouraging physical exercise. Rather than relying on external coaching or structured training programs, parents provided sports equipment for in-home workouts. As a result, it has changed the role of sports for school students, moving from practical goals to personal needs. This shift indicates that students now choose sports activities based on their own needs and enjoyment [19]. With the pandemic over, individual exercise needs can be met through alternative physical activities and innovative support methods [20]. This variety and flexibility can offer more opportunities and motivation for people to maintain their physical well-being and develop positive lifestyle habits [21].

### 2.3. The Impact of Socioeconomic Disparities

The pandemic’s impact on child physical activity and recovery varies across gender, age, ethnicity, and socio-economic status [4]. Notably, low income plays a key role in physical exercise practice. Individuals raised in low-income families have a negative relationship with sports and exercise participation at a young age [22]. Active children before the lockdowns returned to active clubs, while less active children are now even harder to engage, leading to a greater divide in children’s ability and activity levels [4]. Research shows that socioeconomic disparities in physical fitness persist in school settings, with economically disadvantaged students—especially racially minoritized students—performing worse than their peers in physical fitness assessments [5]. The concentration of poverty surrounding schools and the disparities in resource allocation contribute to these inequities, particularly affecting Latin American, American Indian, and Native Hawaiian students [5].

### 2.4. Devaluation of Physical Education

Physical distance during the pandemic threatened the identity of physical education, complicating curriculum coverage and reducing social and emotional support for students [23]. Teachers faced added responsibilities and struggled with frustration, stress, and insecurity while teaching physical education during lockdowns. The online teaching of physical education during the lockdown was uncertain, with some online classes being used to fill “empty slots” in the schedule of classes [24]. After pandemic restrictions ended, many schools returned to pre-pandemic physical education practices, though some activities kept a different pedagogical approach to prevent contagion for a while [17]. However, funding disparities and the lack of mandated physical fitness programs in many U.S. states further exacerbate these issues, limiting access to quality physical education for underprivileged students [5].

The existing literature highlights the detrimental impact of the COVID-19 pandemic on physical fitness, particularly in youth populations, emphasizing declines in activity levels, increased sedentary behaviors, and disparities based on socioeconomic status. Additionally, studies underscore the motivational challenges faced by both students and educators in re-engaging with physical education post-pandemic. While existing studies provide an overview, they lack detailed insights from physical education instructors. This study fills that gap with qualitative insights from educators, offering a nuanced view of these challenges in schools. By combining these perspectives with existing research, the study provides a comprehensive understanding of the evolving landscape of physical fitness education.

## 3. Materials and Methods

### 3.1. Methodological Framework

We used a phenomenological framework and methodology to understand the impact of COVID-19 on physical education in schools and to explore the lived experiences of students and educators during the pandemic. This approach helps us uncover the nuanced ways in which the pandemic has reshaped physical education practices and student engagement. The concept of phenomenology was originally developed by Edmund Husserl and was later expanded by Maurice Merleau-Ponty and Martin Heidegger [25]. Phenomenology is a method of the study of phenomena from the point of view of those who have experienced them in order to describe their essence [26]. As physical education teachers, the participants have directly experienced the impact of COVID-19 on physical education and have interacted with students who shared their experiences. Furthermore, phenomenology utilizes interviews for the collection of data and focuses on the way in which humans experience the world. This study utilizes semi-structured interviews as a method of data collection.

To enhance methodological transparency and rigor, the interview protocols were developed with reference to phenomenological research practices as outlined by Patton and Teherani et al. [26,27]. We employed semi-structured interviews, allowing flexibility in exploring participants’ lived experiences while maintaining a consistent thematic structure across interviews. The initial set of interview prompts was constructed around the study’s central research questions, which aimed to understand the perceived impacts of the COVID-19 pandemic on physical fitness, motivation, and equity within K–12 physical education.

Sample interview questions included:

- “Can you describe any noticeable changes in your students’ physical fitness levels since the COVID-19 pandemic began?”
- “What challenges have you encountered related to student engagement and motivation in physical education classes post-pandemic?”
- “In your experience, how have socioeconomic factors influenced your students’ “participation and access to” physical fitness opportunities during and after the pandemic?”
- “What kind of institutional or administrative support have you received to address these challenges?”

To ensure rigor and minimize researcher bias during data analysis, we employed several strategies consistent with qualitative best practices. First, we used in vivo coding during the initial round of analysis, capturing the precise language and terminology expressed by the participants [28]. This approach enabled us to represent authentically the teachers’ lived experiences without prematurely imposing external interpretations.

Following initial coding, thematic analysis was performed as a second analytical cycle, involving peer debriefing and member checking to validate emergent themes and interpretations. Peer debriefing involved regular discussions within the research team, critically evaluating coding decisions and interpretations of the data. Member checking allowed participants to review and confirm the accuracy and resonance of our interpretations, further enhancing the trustworthiness of our findings [29,30]. Together, these methodological steps ensured a comprehensive, rigorous approach to capturing and analyzing the nuanced experiences of physical education teachers, thereby strengthening the credibility and reliability of the study’s findings.

### 3.2. Data Collection

We recruited 11 participants by contacting them in one of the school districts in Southern California. We used criterion sampling, a type of purposive sampling based on specific criteria or characteristics of the participants, as defined by Patton [27]. The inclusion criteria consisted of physical education teachers in Ventura County, California, who have taught physical education before, during, and after the pandemic. To conduct this study, we relied on semi-structured interviews to collect data. Despite having a pre-planned topic framework, the actual questions for each interview varied slightly, depending on the themes identified by the teachers during the interview.

The interviews took place digitally via Zoom in one-on-one meetings. To protect participants’ privacy, no video was recorded; only audio was used to produce transcripts for coding the collected data. Interviews were transcribed using Zoom’s transcription feature and then by Rev.com. The research team reviewed each transcription for accuracy, with each member listening to the corresponding interview to ensure the highest level of accuracy. To protect the confidentiality of the participants, we agreed not to use any names during the interviews. We wanted the participants to be transparent about their experiences with colleagues and administrators, and their anonymity allowed them to share their experiences without fear of retaliation from their colleagues and/or superiors. We interviewed participants for up to 90 min in exchange for a $50 Amazon gift card.

### 3.3. Participants Description

The participant group consisted of eleven physical education teachers from a single school district in Southern California. The sample included eight females and four males, ranging in age from their early 30s to late 50s. Most participants identified as White Americans, with one participant identifying as Latin American. Most participants had over 10 years of teaching experience, with some exceeding 20 years in the field. The teaching levels represented included elementary (K-6), middle school (grades 6–8), and high school (grades 9–12).

Participants worked in various school contexts, with many teaching in low-income or Title I schools serving predominantly Latin American student populations. A few taught in magnet or more socioeconomically diverse schools, while several emphasized the presence of students from low- to lower-middle-income households, including children experiencing housing insecurity. The socioeconomic status among the teachers themselves ranged from middle-class to upper-middle class. Political affiliations were mixed, with participants identifying as Republican, Democrat, Independent, or unspecified/uncertain, see Table 1 for a list of demographics.

### 3.4. Data Analysis

As part of this study, we chose in vivo coding as our initial coding method which is a code is based on the actual language used by participants in the qualitative data record [28]. We wanted to minimize our own biases and experiences regarding the topic, so we believed that using in vivo coding in the first cycle would enhance the research’s trustworthiness and incorporate the participants’ language into the study.

After the first cycle of coding, we conducted a second cycle of thematic analysis [29,30]. To further enhance the research’s trustworthiness, we employed memoing, peer debriefing, and member checking. We identified seven common themes mentioned by participants: decline in physical fitness post-pandemic, lack of motivation and engagement, challenges with participation and compliance, the impact of socioeconomic disparities, devaluation of physical education, difficulties in assessing physical fitness post-pandemic, and a need for additional resources and support. These themes are discussed in more detail in the findings and discussion sections of the article.

## 4. Results

### 4.1. Decline in Physical Fitness Post-Pandemic

Across multiple interviews, physical education teachers shared that students’ physical fitness levels have significantly declined since the COVID-19 pandemic. Sara said, “Every year since COVID, student fitness has declined”. Andy commented, “I would say it’s been a pretty steady drop in just physical fitness in general, but the pandemic made it worse”.

Before the pandemic, students were generally more active, had better endurance, and were more eager to participate in physical activities. Trevor mentioned, “They don’t have the motivation to try to move. They are not as mobile, and they don’t feel as good”. Meaghan stated, “Students were better organized and had stronger fitness routines”. Andy confirmed, “Students had higher fitness levels and a stronger commitment to physical activity”.

Now, many students struggle with basic exercises like running, stretching, push-ups, and team sports. Andy stated, “Students now showing less stamina, lower flexibility, and weaker endurance compared to five years ago”. Sara said, “Running the mile has become more difficult, with some students struggling to complete it”. Dustin confirmed, “Some kids physically don’t understand how to do basic exercises”.

Teachers have also noticed an increase in sedentary behavior, weight gain, and difficulty in completing fitness assessments. Trevor said, “I don’t think they have changed. I think it kind of reset the bar to even a new low that educators are now struggling to get physical education back. I’ve noticed a big change in my students just in general. They are in worse shape than they were before COVID”. Andrew expressed concern, “We were all in a sedentary lifestyle. It was hard to get people to get up and do something”. In order to fight sedentary behavior, Tamara shared, “I would love for every student to have a Fitbit or activity tracker”.

### 4.2. Lack of Motivation and Engagement

A common theme among teachers is the lack of student motivation to engage in physical education, making it challenging to encourage participation. Trevor shared, “They don’t have the motivation to try to move”. Sara confirmed, “I find it challenging to motivate students who come in with the idea that they hate running or that they aren’t good at it”. Lorna stated, “Some students just stand there and stare blankly or refuse outright”.

Students often make excuses to avoid physical activity, and some refuse to participate altogether. Andy said, “Everything comes down to a doctor’s note or ‘My mom said, my dad said.’ They try to get out of even the most basic exercises”. Oscar mentioned, “There’s a lot more of ‘I can’t’ or ‘I don’t want to’ than before COVID”. Tamara shared concern, “Some just refuse to dress out or participate at all”.

Many seem disengaged, preferring passive activities or socializing over structured exercise. Meaghan complained, “They are so connected to their devices that they struggle to focus on anything else”. Dustin confirmed, “They just want to stand around and be on their phones”. Grace added, “Too many students just sit around and chat instead of playing”.

Some educators feel that student attitudes toward physical education have worsened since the pandemic, making it even harder to foster enthusiasm. Lorna stated, “The bottom line is, the kids just weren’t gonna do it. Most of them wouldn’t even try”. Dustin complained, “Many students avoid physical education entirely, even if it’s mandatory”. Andrew confirmed, “20 to 30% of my students don’t care at all. They don’t want to be here”.

### 4.3. Challenges with Participation and Compliance

Teachers have noticed increased resistance from students when it comes to dressing out for physical education and following participation requirements. Dustin said, “More students outright refuse to dress out or participate”. Andrew complained, “Some students think putting their shirt around their neck counts as dressing out”. Andrew stated, “Physical education participation is an ongoing battle”.

Inconsistent school policies on discipline and compliance make it difficult for educators to enforce participation. Some schools have relaxed their expectations post-pandemic, reducing the consequences for non-compliance. As a result, students who refuse to dress out or participate often face little to no consequences, leading to frustration among teachers. Trevor complained, “We lost a lot of control in the schools”. Andrew confirmed, “Dress-out policies are inconsistently enforced”. Andrew shared, “The school has a dress-out policy, but administrators aren’t enforcing it. Students are supposed to get detention for not dressing out, but that never happens”. Tamara agreed, “We mark tardies and non-dress-outs, but nothing happens”.

### 4.4. The Impact of Socioeconomic Disparities

Physical education teachers observed that students from lower-income backgrounds faced greater challenges in maintaining physical fitness during the pandemic. Trevor shared, “Students at or below the poverty level had the biggest struggle. These students were already at a disadvantage, and the pandemic made it worse”. Meaghan said, “We have a wide range of socioeconomic diversity, which impacts student participation in fitness programs”. Grace added, “Lower-income kids play at the park or ride bikes, but don’t have the same structured athletic training”.

Limited access to outdoor spaces, lack of structured physical activity at home, and poor nutrition contributed to lower fitness levels. Grace complained, “Students didn’t have enough space to stretch or move properly”. Dustin added, “Most students don’t have access to sports or structured fitness outside school. Some students have never had real nutrition education”.

Many students from lower-income families had fewer opportunities to engage in extracurricular sports or fitness programs compared to their wealthier peers. Sara said, “Wealthier students participate in organized sports, while lower-income students may lack access”. Meaghan added, “Students with the financial means traveled out of state to play in tournaments”. Grace confirmed, “Kids from wealthier families are more likely to be on organized sports teams”. Tamara concluded, “Low-income families often can’t afford organized sports”.

Teachers noticed a significant contrast between students who had access to private sports training and those who did not. Andy shared, “Club sports are expensive, so lower-income students have fewer chances to develop athletic skills”. Oscar said, “A lot of kids don’t participate in extracurricular activities. Their skill levels are noticeably lower”.

### 4.5. Devaluation of Physical Education

Teachers believe that physical education is often overlooked in favor of academic subjects, particularly after the pandemic, when the emphasis was on catching up in math and literacy. Trevor complained, “Schools focus on math and reading, but physical education is not a priority. Physical education needs to be treated like math and language arts, with daily sessions and real accountability”. Andrew added, “Physical education is always on the back burner compared to core subjects”. Andy concluded, “Physical education is undervalued”.

School administrators tend to prioritize core subjects, which reduces support for physical education programs. Trevor shared, “No, my administrators do not value physical fitness programs”. Andrew added, “We haven’t seen an administrator at physical education in months. We ask for meetings, and no one comes”. Andy said, “Physical education suffers from inconsistent policies and lack of administrative support. We ask for more support, but nothing changes”.

Some teachers are frustrated by the lack of funding and resources dedicated to physical education programs. Dustin complained, “We have six kids per station, sometimes seven, and not enough equipment”. Tamara expressed concern, “Teaching swimming to 54 kids with no lifeguard is a disaster waiting to happen”. Meaghan concluded, “If we had more funding and smaller class sizes, every student would benefit”.

### 4.6. Difficulties in Assessing Physical Fitness Post-Pandemic

Many teachers have faced challenges in assessing students’ physical fitness levels due to inconsistent participation and effort. Andy shared, “Students are graded on effort, not performance, but even then, some don’t try”. Trevor added, “Kids today can’t do what kids in the ’90s and ’80s did”.

Students often underperform on fitness tests, making it difficult to gauge their true physical abilities. Sara commented, “Running the mile has become more difficult, with some students struggling to complete it”. Dustin said, “Some kids physically don’t understand how to do basic exercises”. Tamara shared, “Some kids don’t even know how to jog anymore”.

Consequently, teachers have had to adjust their assessment strategies, sometimes lowering expectations to account for the post-pandemic decline in fitness. Trevor concluded, “In reality, most students fail to meet state fitness standards”. Lorna said, “I have lowered my standards some. I don’t ask them to do certain things anymore”. Tamara shared, “I tell them, ‘I don’t care if you can’t run. Let’s start with walking”.

### 4.7. Need for Additional Resources and Support

Teachers believe that increased funding for physical education could help re-engage students and promote better fitness outcomes. Meaghan said, “If we had more funding and smaller class sizes, every student would benefit”. Tamara added, “More funding would allow for better tracking, equipment, and individualized support”. At the same time, Drew expressed concern that “Investments in new fitness equipment have helped but have not fully solved the problem”.

Additional staff support, such as assistants or specialized fitness trainers, could address challenges related to student motivation and engagement. Tamara complained, “We have 54 kids per class. It’s impossible to give individual attention when you have that many students”. Lorna agreed, “Individual instruction is difficult with 40+ students per class”. Dustin summarized the current situation, “We ask for more support, but nothing changes”.

More institutional backing from administrators and policymakers is needed to emphasize the importance of physical education and encourage student participation. Oscar complained, “I think our government currently isn’t doing a good job of making physical education important. If kids don’t hear about it, they don’t internalize its value”. Meaghan agreed, “I don’t know if I’m knowledgeable enough to answer that, but I haven’t seen enough emphasis on physical education from the government”. Dustin elaborated further, “I don’t think the government values physical education the way they should. They say they support it, but their actions don’t reflect that”. Tamara concluded, “The government makes rules about physical education, but they’re not meaningful. They don’t actually understand what we deal with in schools”.

## 5. Discussion

### 5.1. Reinterpretation of the Study

**Figure 2:**
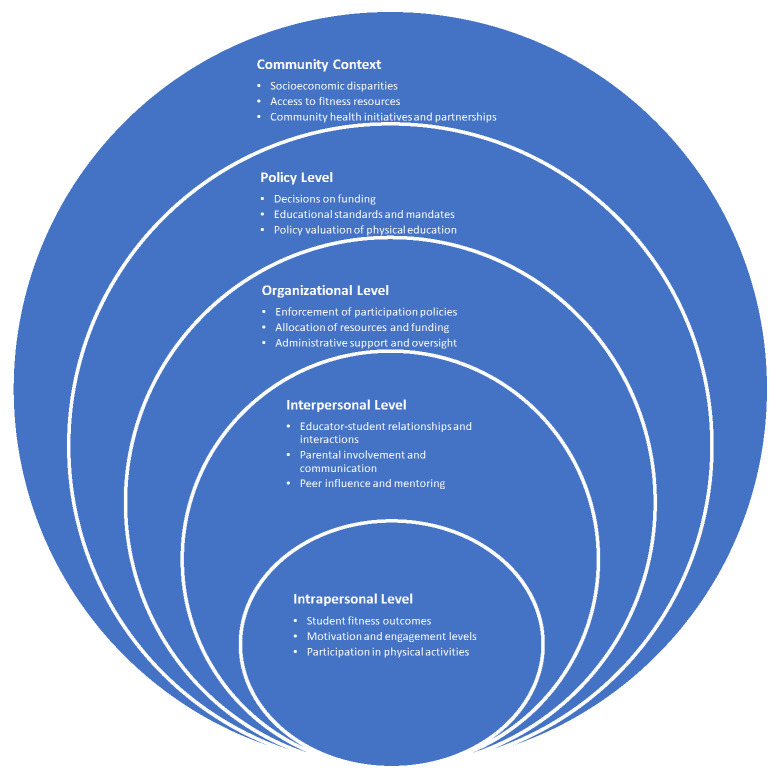
New socioecological model for physical fitness.

This study demonstrates that the COVID-19 pandemic has significantly affected student physical fitness, motivation, engagement, and access to physical education. Physical education teachers’ experiences, analyzed through the Social Ecological Model of Health Behavior, demonstrate the influence of intrapersonal, interpersonal, organizational, community, and policy factors on the health behaviors of U.S. K-12 students [6]. See Figure 2 for an illustration of our new model for physical fitness.

At the intrapersonal level, students demonstrated substantial declines in fitness and motivation, reflecting internal psychological and physical barriers exacerbated by the pandemic [2,3]. Educators specifically highlighted the establishment of a new, lower baseline of sedentary behavior and decreased engagement, underscoring the profound personal-level disruption experienced by students. As Trevor explained, “I think it kind of reset the bar to even a new low”.

Interpersonally, educators described heightened challenges in motivating students and fostering engagement, emphasizing the critical role of social interactions within physical education. “They don’t have the motivation to try to move,” according to Meaghan and Sara with Lorna adding, “some students just stand there and stare blankly or refuse outright”. Teachers’ efforts to reconnect with students revealed the significance of peer relationships and educator–student interactions in sustaining student motivation and participation in physical activities. Additionally, the increased reliance on parental support during remote learning highlighted the importance of familial influence on students’ physical activity habits [19].

At the organizational level, the inconsistent enforcement of school policies emerged as a major barrier to student participation and compliance. Teachers identified how unclear and unevenly applied institutional policies significantly hindered their ability to enforce participation requirements, further demotivating students and diminishing the overall effectiveness of physical education programs. “Dress-out policies are inconsistently enforced” bemoaned Andrew with Tamara agreeing that “we mark tardies and non- dress-outs, but nothing happens”. This finding reveals a critical intersection between administrative practices and student behavior, emphasizing the need for cohesive institutional strategies [24].

Community-level disparities were particularly evident through socioeconomic factors shaping students’ access to structured physical activity and sports programs. Teachers explicitly linked socioeconomic status to reduced participation opportunities, inadequate nutritional resources, and limited access to private fitness options, reinforcing existing research on the amplifying effects of community contexts on educational and health inequalities [4,5]. As Trevor shared, “Students at or below the poverty level had the biggest struggle. These students were already at a disadvantage, and the pandemic made it worse”. As Grace highlighted that lower-income students “have fewer opportunities to develop athletic skills”. Andy further explained that the reason for this is that “club sports are expensive, so lower-income students have fewer opportunities to develop athletic skills”.

Finally, at the policy level, the undervaluation of physical education programs by administrators and policymakers was profoundly felt by educators. This systemic marginalization manifested in insufficient funding, inadequate resources, and a general lack of institutional support, underscoring how broader educational policies directly affect the quality and accessibility of physical education [23]. These policy shortcomings highlight the necessity for systemic reforms to strengthen institutional backing and policy coherence at state and national levels. By explicitly linking these findings to each layer of the Social Ecological Model, this analysis not only deepens the understanding of the multifaceted impacts of COVID-19 but also provides a clear, structured basis for targeted interventions across multiple ecological levels to rejuvenate physical education and student health outcomes. See Table 2 for a visual representation of this section.

### 5.2. Contribution to Knowledge

The findings of this study not only align with existing literature on the negative impacts of COVID-19 on student fitness and engagement but also provide novel insights and unexpected nuances not fully addressed in prior research. Consistent with prior studies, we observed substantial declines in student physical fitness, notably endurance, flexibility, and strength [2,3]. However, the qualitative depth of our data highlights an alarming new baseline of sedentary behavior among students, which educators described as “resetting the bar” to unprecedented lows, revealing a potentially enduring impact beyond the initial disruptions identified by Chen et al. [1].

A particularly novel finding in our study was the intensified resistance from students towards compliance with traditional physical education requirements, such as dressing out for class. While existing research has acknowledged decreased student engagement post-pandemic [16,17], our study uniquely identifies inconsistent enforcement of participation policies as a significant barrier exacerbating student resistance. This highlights an organizational-level challenge that suggests the need for policy coherence within schools to effectively address student compliance and engagement.

Furthermore, while socioeconomic disparities in physical education access and participation have been documented [4,5], our qualitative findings provide deeper insight into specific mechanisms exacerbating these disparities. Teachers explicitly highlighted the stark contrast between students with and without access to private club sports and structured fitness programs, an issue less emphasized in existing literature. This distinction underscores the urgent need for public school systems to bridge the resource gap through more inclusive, community-oriented programs.

An unexpected insight emerging from our data was the degree of frustration educators experienced regarding institutional undervaluation of physical education, which extended beyond funding and resources to include perceived neglect from administrators and policymakers. Although existing literature has noted physical education’s marginalization [23,24], the explicit emotional and professional toll documented in our findings provides a compelling argument for systemic cultural shifts within educational institutions. Collectively, these novel insights and nuanced understandings expand upon the existing body of research by providing a richer, more detailed picture of the pandemic’s multifaceted impacts. This enhanced understanding not only validates prior research but also sets a critical foundation for targeted and innovative approaches to revitalizing physical education in the post-pandemic landscape.

### 5.3. Limitations

While this study offers valuable insights into the impact of the COVID-19 pandemic on physical education from the perspective of K-12 educators, several limitations must be highlighted. First, the findings are based on a small, geographically localized sample of 11 physical education teachers from a single school district in Southern California. Despite their diverse teaching levels and school contexts, the regional focus limits the generalizability of the findings to other states. At the same time, we would like to highlight that a relatively small sample size of 11 participants is sufficient to reach data saturation. For example, the study conducted by Hennink & Kaiser [31] suggests that 9–17 interviews are sufficient to reach saturation. Another study states that a qualitative study needs only 6–12 interviews. Additionally, although the study is limited to Southern California, we believe that it is appropriate to begin with California since it has a diverse student body, and policies proposed in California are frequently replicated in other states.

Second, although the study employed rigorous methods such as in vivo coding, peer debriefing, and member checking, researcher interpretation still influences thematic analysis. Despite efforts to minimize bias, the findings inevitably reflect the subjective perspectives of both participants and researchers.

Finally, the study exclusively focused on the perspectives of educators. While their insights are crucial, future research could benefit from including the voices of students, parents, and administrators to provide a more comprehensive view of the post-pandemic physical education landscape. Despite these limitations, this study contributes valuable qualitative evidence to the growing body of literature on how the COVID-19 pandemic reshaped school-based physical education.

### 5.4. Policy Implications

This study reveals a need for policy interventions at multiple levels to address the post-pandemic challenges facing physical education in K-12 schools. Due to the widespread decline in student fitness and motivation, policymakers should reinstate physical education as a core academic subject, as they do for math and language arts. The state and local education agencies should establish minimum requirements for physical education at all grade levels and enforce uniform participation and assessment standards.

Underfunding of physical education programs must be addressed. There is a need to make investments in updated fitness equipment, outdoor and indoor facilities, and professional development for teachers. It is also important for schools to receive funding to reduce class sizes in physical education so that students can receive more individualized instruction and safer supervision, particularly in high-risk environments like swimming lessons. Grants from the federal and state governments could be expanded to prioritize schools serving low-income and racially minoritized students who are disproportionately affected by fitness disparities.

Dress-out and participation policies are not consistently enforced, which has had a significant negative impact on physical education instruction. The school board and administrators should clarify behavior guidelines and uniformly implement them in physical education settings so that teachers can exercise their authority. As part of this effort, school leaders should attend classes, respond to teacher concerns, and invest in physical education by ensuring that noncompliance has appropriate consequences.

Education and public health policies at the state and national levels must incorporate a health equity lens into the design of physical education standards. The pandemic has widened disparities in physical fitness across racial and income groups. There is a need for policies that ensure that historically underserved student populations receive adequate physical education and health resources, including Latin American, American Indian, Alaska Native, and Native Hawaiian students. The process may involve the collection of disaggregated data, the support of culturally relevant fitness programs, and the integration of physical education goals into broader educational equity programs.

To address the post-pandemic challenges identified in this study, specific actionable recommendations for school administrators and policymakers are essential. First, school administrators should prioritize consistent enforcement of physical education participation policies, as inconsistent discipline was found to significantly hinder student engagement. Schools could establish clear, unified guidelines, paired with regular training sessions for educators and staff, to reinforce consistency and accountability. Second, administrators should actively invest in targeted initiatives that mitigate socioeconomic disparities affecting student fitness. Successful interventions include implementing comprehensive school-based physical activity programs accessible to all students regardless of economic status, such as the Active Schools initiative, which has demonstrated effectiveness in promoting equitable physical activity opportunities [5]. Additionally, partnering with local community organizations to provide low-cost or free after-school fitness programs can significantly bridge existing resource gaps.

Third, policymakers should consider mandating minimum physical education standards at the state level, coupled with dedicated funding streams. For example, California’s Physical Education Model Content Standards provide a robust framework that could be universally adopted or adapted to ensure structured, high-quality physical education across all districts. Policymakers might also explore successful models like the Carol M. White Physical Education Program (PEP), which historically supported innovative approaches to physical education and has positively impacted student fitness outcomes nationwide. Finally, enhancing institutional support for physical education through sustained professional development is crucial. Policymakers and administrators should allocate funds specifically for ongoing professional training, enabling educators to stay updated with evidence-based practices and innovative approaches to physical education, such as blended or gamified instructional models shown to enhance student motivation and participation [17]. Collectively, these actionable recommendations, grounded in successful precedents and best practices documented in existing research, provide clear, strategic paths toward addressing the systemic issues uncovered by this study, ultimately fostering improved health and equity outcomes in post-pandemic physical education contexts.

### 5.5. Generalizability and Future Research

The findings of this study are derived from a localized sample of physical education teachers within one school district in Southern California. While this geographic specificity provides rich qualitative insights into the experiences and challenges faced within this context, it also inherently limits the generalizability of the results to broader populations and different educational contexts. The particular socioeconomic, cultural, and policy conditions of Southern California schools may differ significantly from those in other regions, impacting the transferability of our findings.

Future research would benefit from examining the impacts of the COVID-19 pandemic on physical education across diverse geographic locations and varied demographic contexts. Comparative studies between urban, suburban, and rural schools, as well as between regions with different levels of funding and policy support for physical education, would enrich our understanding of both universal challenges and context-specific issues. Additionally, studies that specifically include diverse demographic groups, such as varying ethnic, racial, and socioeconomic student populations, would further clarify how intersecting identities and structural conditions influence physical fitness outcomes post-pandemic. Such expanded studies could validate, challenge, or deepen the findings of this research, offering a more comprehensive and nuanced understanding of the pandemic’s long-term impacts on physical education and student health across broader populations. This broader approach is essential for developing tailored and effective policy responses capable of addressing diverse needs and fostering educational equity nationwide.

Future studies should include perspectives from students, parents, school administrators, and community stakeholders, not just educators. Student narratives could shed light on the psychological and motivational changes. Longitudinal studies are essential to track changes in student physical fitness, motivation, and engagement over time. Research following student cohorts over several years could reveal how fitness levels recover or decline further and which interventions are most effective in restoring pre-pandemic physical health and activity levels. Finally, large-scale quantitative studies could complement qualitative findings by identifying trends in physical fitness assessment scores across various demographic groups.

## 6. Conclusions

The semi-structured interviews reflected seven key findings. The first finding revealed that there has been a major decline in physical fitness post-pandemic, with students being much more active before COVID-19. The second finding centered on a lack of motivation and engagement, with teachers struggling to increase participation and full engagement. Thirdly, there were challenges with participation and compliance among students, such that students did not dress to exercise or engage fully in exercises. Our fourth finding revealed the deleterious consequences of low socioeconomic disparities, with students with lower socioeconomic status struggling to engage as well as their peers. Fifthly, there was a general devaluation of physical education compared to academic subjects. Our sixth finding revealed that, because of state practices, there were difficulties in assessing physical fitness post-pandemic. Lastly, physical education teachers wanted what they considered necessary additional resources and support in order to increase engagement in physical education.

This study makes several critical contributions to the existing literature on the impacts of COVID-19 on K–12 physical education by providing rich qualitative insights from educators directly involved in post-pandemic recovery efforts. Through an in-depth exploration guided by the Social Ecological Model [6], the research highlights a comprehensive spectrum of challenges, from intrapersonal declines in student fitness and motivation to broader organizational and policy-level shortcomings. Uniquely, the findings reveal not only the expected deterioration in student physical fitness but also the establishment of a concerningly lower baseline of physical activity and increased student resistance to structured participation. This resistance was significantly linked to inconsistent institutional enforcement of participation policies, a factor previously under-examined in the literature [16,17]. Additionally, the explicit identification of private club sports as a key differentiator in students’ fitness access underscores a specific socioeconomic mechanism intensifying inequities, thus extending the conversation on disparities documented in prior research [4,5].

Furthermore, by capturing educators’ personal and emotional responses to perceived institutional undervaluation, this study adds a nuanced layer to the discourse on educational policy and practice post-COVID-19 [23,24]. Such insights not only confirm existing knowledge but deepen the understanding of how institutional culture and policy intersect to shape physical education outcomes. Ultimately, this research emphasizes the urgent need for systemic, comprehensive reforms—highlighting specific strategies such as increased funding, policy coherence, and resource allocation—to effectively address and rectify the profound impacts of the pandemic on student health and educational equity.

## Figures and Tables

**Table 1:** Participant demographics.

Pseudonyms Selected by Participants	Gender	Age	Race	Teaching Experience	Grade Levels
Lorna	F	50–59	White	34	Middle School
Trevor	M	40–49	White	5	High School
Andrew	M	40–49	White	17	High School
Sara	F	40–49	White	20	Elementary
Andrew	M	40–49	White/Black	18	High School
Grace	F	30–39	White	9	Elementary
Tamara	F	50–59	White	23	High School
Dustin	M	40–49	White	12	High School
Meaghan	F	40–49	White	17	High School
Andrew	M	20–29	White	3	Elementary
Oscar	M	40–49	Hispanic	21	Elementary

**Table 2:** Table summarizing key findings, potential interventions, and anticipated outcomes across ecological levels.

Ecological Level	Identified Issues	Potential Intervention Strategies	Anticipated Outcomes
Intrapersonal	Declined fitness levels; reduced motivation	Personalized fitness programs; enhanced tracking methods; motivational strategies (gamification)	Improved student fitness, increased motivation and engagement
Interpersonal	Lack of student motivation and engagement; limited parental involvement	Enhanced communication with families; peer-based mentoring programs; professional development for teachers on motivational techniques	Increased student engagement; stronger educator–student relationships; improved parental support
Organizational	Inconsistent policy enforcement; inadequate resources	Standardizing and clearly communicating participation policies; consistent administrative oversight; increased funding and resource allocation	Enhanced compliance with physical education policies; equitable resource distribution; improved student participation
Community	Socioeconomic disparities limiting access to fitness resources	Community partnerships to offer accessible after-school fitness programs; community-based nutrition and fitness education initiatives	Reduced fitness and participation disparities; improved community health and wellness outcomes
Policy	Systemic undervaluation of physical education; insufficient funding	Mandated minimum standards for physical education; dedicated funding streams; policy advocacy and awareness campaigns	Increased institutional support; enhanced valuation and prioritization of physical education; equitable and sustainable fitness programs

## Data Availability

The datasets presented in this article are not readily available because the data are part of an ongoing study. Additionally, the data are qualitative in nature and should be kept confidential. Requests to access the datasets should be directed to the first author.
